# Problems with tense marking in children with specific language impairment: not how but when

**DOI:** 10.1098/rstb.2012.0401

**Published:** 2014-01-19

**Authors:** Dorothy V. M. Bishop

**Affiliations:** Department of Experimental Psychology, University of Oxford, Oxford, UK

**Keywords:** specific language impairment, grammar, past tense, morphology, aphasia, inflections

## Abstract

Many children with specific language impairment (SLI) have persisting problems in the correct use of verb tense, but there has been disagreement as to the underlying reason. When we take into account studies using receptive as well as expressive language tasks, the data suggest that the difficulty for children with SLI is in knowing *when* to inflect verbs for tense, rather than *how* to do so. This is perhaps not surprising when we consider that tense does not have a transparent semantic interpretation, but depends on complex relationships between inflections and hierarchically organized clauses. An explanation in terms of syntactic limitations contrasts with a popular morpho-phonological account, the Words and Rules model. This model, which attributes problems to difficulties with applying a rule to generate regular inflected forms, has been widely applied to adult-acquired disorders. There are striking similarities in the pattern of errors in adults with anterior aphasia and children with SLI, suggesting that impairments in appreciation of when to mark tense may apply to acquired as well as developmental disorders.

## 1. Introduction

When children first learn to talk, they don't just imitate the speech they hear: their output reflects limitations of their immature language. Verbs are a particularly rich source of errors. Sometimes we see bare stem errors, where the inflection is simply omitted, as in ‘John go there’ or ‘Yesterday Daddy run the marathon’. In addition, we may see overregularization of an irregular verb, such as ‘I runned home’ or ‘Mummy drived her car’. Both types of error have stimulated theorizing about the underlying nature of the child's grammatical difficulties, but despite many years of research, there is still debate as to their origins.

Problems with verb inflections are seen in typical development and are also a striking feature of both developmental language disorders [1] and some types of acquired aphasia [2]. My main focus here is on English-speaking children with specific language impairment (SLI), a condition that is diagnosed when language is out of step with other aspects of development for no obvious reason. Encouraged, however, by the integrative spirit of this special issue, I briefly consider whether insights from the study of children might also help us to understand abnormal use of verbal inflections in acquired aphasia.

## 2. How is tense acquired?

**Figure 1.**
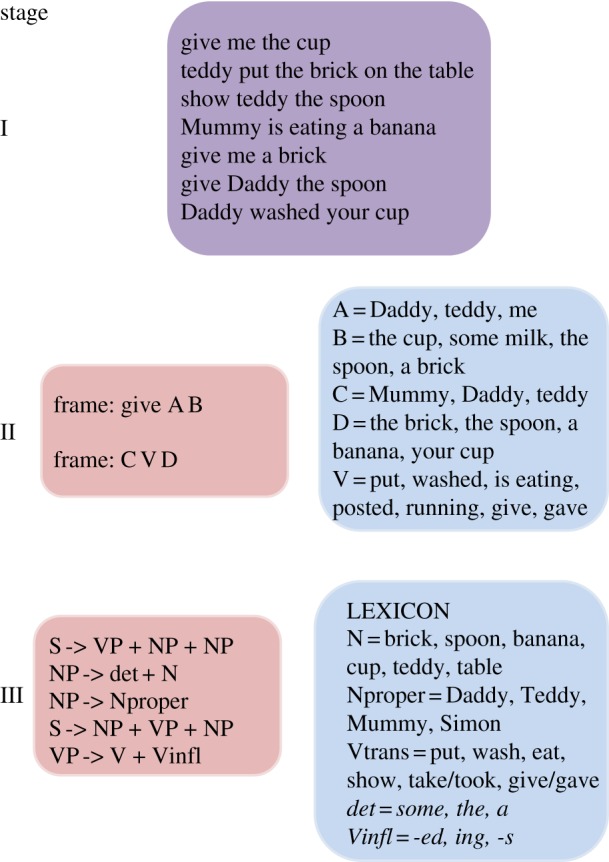
Stages in acquisition of syntax. Initially, rote-learned phrases (purple) predominate (stage I), but as learning proceeds (stage II), there is identification of sentence frames (pink) containing specific types of words (blue). Gradually, knowledge becomes more abstract (stage III), with formation of a lexicon containing phonological forms together with their meanings and syntactic classes, and recognition of phrase structures into which lexical items can be slotted.

Figure 1 shows a schematic sequence for acquisition of grammar inspired by Edelman & Waterfall [3] and Tomasello [4]. Stages are shown to illustrate the kinds of internal representations that predominate at different points in development, but transition from one to the next is assumed to be gradual.

As the child hears more and more language, patterns are identified, so that lexical items can be grouped into syntactic categories that can occupy specific slots in sentence frames (stage II). For instance, the child who hears ‘Mummy is eating’, ‘Daddy is coming’, ‘Mummy is waiting’, ‘Baby is eating’ and so on, will start to categorize Mummy, Daddy and Baby together as the same kind of word. The presence of inflectional endings, such as -ing, as well as co-occurrence with other high-frequency words, such as determiners or auxiliaries, can act as important cues to syntactic category.

Gradually, the child will start to recognize sequential dependencies between abstract units and begin to operate with larger units, such as Noun Phrase and Verb Phrase; this allows a move away from rote-learned utterances so that novel word sequences can be generated [5]. Part of this learning is the recognition that the same verbs can occur with or without suffixes such as /t/ (as in ‘washed’), /d/ (as in ‘robbed’) or /id/ (as in ‘posted’). Once the morphological status of these inflections is appreciated, lexical storage can become more efficient, because only the verb stem need be stored, and inflectional paradigms can be set up. Inflected forms can then be assembled by rule, with the abstract past tense marker, -ed, converted to the appropriate phonological form depending on the verb's final phoneme.

Suppose, however, that the child has auditory difficulties that make it difficult to perceive a morpheme, such as past tense -ed, which in English is often unstressed and not perceptually salient. Leonard [6] proposed just such an account—the ‘surface hypothesis’—to explain SLI. If the child frequently fails to perceive the inflection, then learning of the past tense rule will be delayed, because it depends on acquiring a critical mass of inflected and uninflected verb forms, so that the pattern can be detected.

A related idea is that learning of tense could be impaired by problems with phonological segmentation. The child who has no difficulty distinguishing, for instance, ‘walk’ and ‘walked’, may nevertheless treat both words as unanalysed wholes and fail to recognize their component phonemes. This has been termed the ‘phonological deficit hypothesis’ [7]. If the child fails to parse an inflected verb into stem and inflection, then lexical representations of verbs may remain at stage II and this will impair ability to learn the significance of inflections.

A similar outcome could arise if, despite identifying phonological segments, there was a problem in identifying regularities in the input, so that Verb + -ed was not recognized as a recurring pattern. Gopnik & Crago [8] suggested that such an account might explain problems with grammatical features in members of a three-generational family affected with an inherited form of SLI. Specifically, they proposed that affected individuals ‘…have a learning mechanism that sees each word as an independent item that must be learned and entered into a lexicon that specifies its grammatical properties and meaning’, and they ‘do not have the normal language-learning mechanism…that would allow them…to construct inflectional paradigms on the basis of regularities hypothesised from the observed linguistic evidence’ (p. 47). Others, coming from a very different perspective, have proposed rather similar ideas without assuming that the problem is specific to grammar. Rather, it has been suggested that inability to detect recurring syntactic patterns might be part of a broader difficulty with statistical learning [9]. We can see, then, that there are several reasons—perceptual limitations, poor phonological segmentation and a deficit in identifying linguistic regularities—all of which could lead to delays in identifying patterns of regular inflection.

According to the timeline shown in figure 1, irregular past tense verbs would only be identified as tensed forms once the child had isolated the -ed morpheme from regular verbs and identified some of the syntactic and semantic features associated with it. These same features could then be stored in the lexicon with the irregular verb. Thus, the child would start out at stage II with a single, lexical means of representing verbs, inflected or not, with syntactic features becoming established at stage III, as more and more regular verbs are encountered.

But how does the child identify the conditions under which inflections are obligatory? Tense is a particularly complex feature of grammar, for two reasons. First, the functional significance of a tense inflection is much harder to deduce than that of a semantically transparent inflection such as noun plural -s. Although -ed indicates past tense of a verb, it does not always correspond to past time: for instance, we say ‘I saw him **jump**’ or ‘I wanted to **escape**’ rather than ‘*I saw him **jumped**’ or ‘*I wanted to **escaped**’, even though the jumping or escaping is in the past. In a passive construction, we may use an -ed inflected verb for a current event or future event, e.g. ‘The dog is being/will be **groomed** by the man’. Second, for most inflections, there are reliable local cues that determine grammaticality; for instance, consistency of verb–subject agreement in number makes it easy to learn that some sequences such as ‘they comes’ or ‘he am going’ are ungrammatical. In English, local cues are not, however, a reliable guide to the contrast between finite (tensed) and infinite (bare stem) verb forms. First, in emphatic, question and negative sentences, tense is marked on an auxiliary verb rather than the main verb, and in some contexts, notably questions, there may be distance between the tensed auxiliary and the stem, e.g. ‘**Did** the boy over there **jump** in the pool?’. Even more complex is the system of verb complementation, where a clause is treated like the object of a verb. English allows for both finite and non-finite complements: a sequence such as ‘John jump’ is acceptable in the context ‘I made John jump’, but not in ‘*John jump yesterday’; ‘John jumped’ follows the opposite pattern: acceptable as ‘John jumped yesterday’ or ‘I know John jumped yesterday’ but not as ‘*I made John jumped yesterday’. Thus, to master tense the child must establish the relationship between clauses in a multi-clause utterance, and then relate these to small sublexical units (inflections).

## 3. How is tense marking applied once language has been learned?

**Figure 2.**
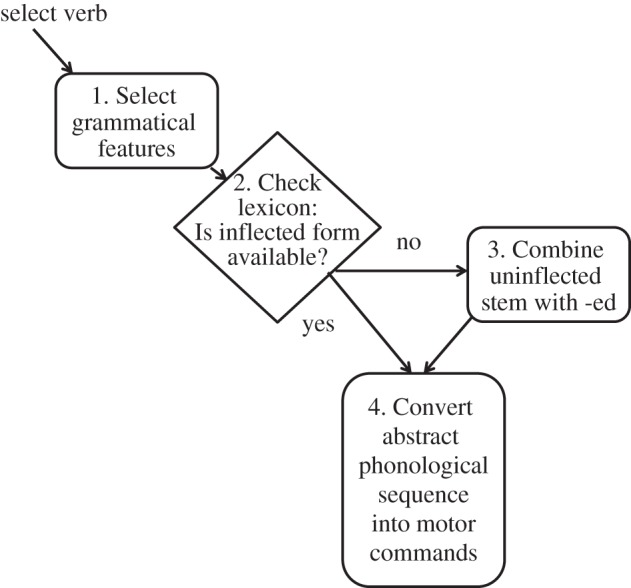
Stages in production of tense-inflected verbs.

Figure 2 shows stages in producing a tense-inflected verb. This model, which is based on the Words and Rules view of language processing, distinguishes between processing of irregular verbs, which are represented as inflected forms in the lexicon, and regular verbs, where the regular form is assembled by rule [10]. This separation of processing routes is not universally accepted [11] but will serve the purpose here of indicating stages in processing that could be affected in a child who made errors in inflecting verbs. Problems in marking inflections will arise if there is a failure of learning at any stage—if knowledge of syntax is deficient (step 1), if lexical representations of irregulars are missing (step 2) or if knowledge of the past tense -ed rule is shaky (step 3). A key point, however, is that grammatical errors need not be a sign of poor *learning*: a person may make errors in the course of *computing* a linguistic representation: at the stage of assigning grammatical features (step 1), looking up an entry in the lexicon (step 2), combining a stem with an inflection (step 3) or assembling a motor programme to articulate a phonological sequence (step 4).

We may draw an analogy with a person presented with a sequence of arithmetic problems in rapid succession. Even if she was fully familiar with basic arithmetic, she would make mistakes if the presentation rate made it hard to solve one problem before the next one came along. In the context of verb inflections, the notion is that cognitive resources are consumed by the process of generating a sentence in real time, and tense inflections may be particularly vulnerable when resources are limited [12].

## 4. Evaluating the evidence

We turn now to consider evidence for different explanations for children's problems with tense marking. This takes three forms: performance on production tasks, performance on receptive language tasks and correlational evidence.

### (a) Past tense inflections in SLI. Language production

#### (i) Elicitation tasks

**Figure 3.**
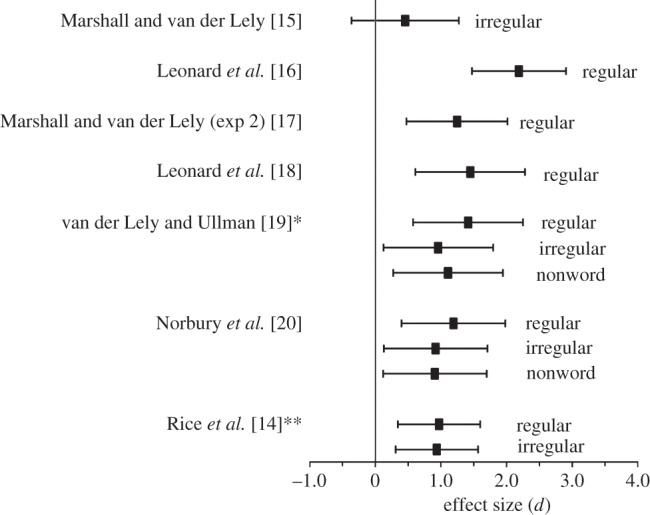
Effect sizes for marking of past tense (irregular form or -ed) in SLI. Inclusion criteria for a study were (*a*) children with SLI compared with younger children matched on a general measure of language level, (*b*) use of an elicitation task; (*c*) sample size of at least 12 per group. The centre point of each bar is the mean effect size in standard deviation units (Cohen's *d*), and the fins show the lower and upper 95% confidence limits. *Compared with youngest language-matched control group; **averaged across seven test occasions.

In a typical elicitation task [13], an introductory sentence is used, such as ‘Every day I eat some chips. Just like every day, yesterday I….’ with the expected completion ‘ate some chips’. This type of format has been used with regular and irregular verbs, and with novel (nonsense) verbs. Comparison of these verb types is complicated by the fact that an incorrect response to an irregular verb can take the form of a bare stem or an overregularization (e.g. ‘falled’). As noted by Rice *et al.* [14], children with SLI produce correctly inflected irregular verbs at a similar rate to typically developing children who are 2 years younger. However, they are significantly less likely than these younger children to produce overregularizations, instead producing a high proportion of bare stems. If we focus only on whether tense is marked in obligatory contexts, children with SLI show a substantial impairment even relative to younger control children who are similar on measures of overall language ability (‘language-matched’ controls). This is a particularly stringent comparison: where age-matched controls are used, the observed deficits are even more striking, but one often sees ceiling effects in the controls. Figure 3 shows a forest plot, depicting effect size when comparing past tense marking by children with SLI relative to language-matched controls. For this plot, irregular verbs were treated as correctly inflected if the child used either the correct irregular form, or an overregularization with -ed (e.g. ‘runned’). The first point to note is that these data confirm that problems with past tense inflections are a striking feature of children with SLI: the effect sizes are typically close to 1.0. A study with a smaller effect size compared 13-year olds with SLI with language-matched controls some 7 years younger [15]. The second point is that the deficit is apparent for regular and irregular verbs: for both verb types, children with SLI are more likely than language-matched controls to produce bare stems in contexts where an inflected form is required. The difference between regular and irregular verbs varies from study to study; this may well depend on verb-specific effects [21]. We know that children's ability to produce inflections is influenced by factors such as the frequency of the stem, frequency of the inflected form, phonological composition of the stem and inflected form and semantic aspects of the verb [16,17,22–24]. Although recent studies have attempted to control for such factors, we can never be sure that we have equated items sets on all relevant variables [21]. Data in this field are seldom analysed to test for item-specific effects, but in one study where this was done, an effect of verb regularity that was significant by subjects was not significant by items [19]. In other words, the difference between irregular and regular verbs was not consistent across verbs, but depended on the specific words used.

Difficulties with applying the -ed inflection to verbs were shown to be syntax specific in an ingenious experiment by Leonard *et al.* [18]. They showed that children with SLI were more likely than language-matched controls to omit -ed on verbs in both past tense and passive participle contexts (e.g. ‘the spoon got washed by the bunny’), but their performance was significantly better in the passive sentences.

A further point to note is that children with SLI are able to apply an appropriate inflection in a rule-based fashion. Although their performance on elicitation tasks is very poor relative to controls, they nevertheless inflect some verbs correctly, even when the verb is an unfamiliar nonsense form: for instance, re-analysis of data from the study by Norbury *et al.* [20] revealed that all children with SLI produced a correctly inflected form for at least one nonword, and the mean score was 5.17 of 16 nonwords correct (s.d. = 4.00). Furthermore, overregularizations, although uncommon, were seen for irregular verbs (see also [25]).

#### (ii) Sentence repetition

In a repetition task, the phonological form is provided and the only requirement is to copy it. Such tasks place minimal demands on grammatical or lexical processing, and it might seem that they should be difficult only if there are deficits at the output phonological stage. However, the simplicity of the task is rather misleading, and it is well established that ability to repeat a sentence is related to ability to process its syntactic structure [26].

Dalal & Loeb [27] were interested in whether the perceptual salience of an inflected verb affected accuracy of children's repetition. They studied 10 children with SLI aged from 4 to 6 years. Children were asked to repeat 10 sentences with sentence-internal verbs, e.g. ‘She skated on the ice’, and 10 sentences of the same length, with the same verbs in final position, e.g. ‘The tall thin girl skated’. All verbs had a syllabic -ed ending. Accuracy was significantly higher for the sentence final verbs, and the authors concluded that the results supported Leonard's surface account of morphological errors [6]. However, misperception of inflectional endings could not account for all the data: 46% of errors involved producing either the wrong verb or a verb with a present progressive tense marking, and 21% involved verb omission, leaving only 32% of bare stem errors. Also, the 10 children had been selected as doing poorly on production of verb inflections in a pretest, yet three of them made no errors on the repetition task, and only four children made three or more errors in 20 items, indicating that repetition is easier than elicited production. No controls were tested, and so it is not possible to tell whether the effect of verb salience was an unusual feature of SLI or a normal developmental phenomenon. Overall, this study shows that repetition of an inflected verb is affected by sentence position, but it does not demonstrate that perceptual difficulties are an adequate explanation for problems with verb inflections in SLI.

#### (iii) Written language

Written language is of interest because, although it develops out of spoken language, it places different demands on processing. On the one hand, we might expect children to do better in producing inflected forms that they know, given that phonological production and poor perceptual ability are unlikely to be an issue, and there is less time pressure than with speech. On the other hand, if a child is still learning to be literate, combining the task of retrieving spellings and formulating sentences with computing correct inflectional endings may be more taxing than in talking. Windsor *et al.* [28] obtained evidence that production of inflections in written language was indeed particularly difficult for children with SLI. They compared written and spoken language samples from 10- to 12-year olds with SLI with those of both age-matched controls and language-matched controls some 2 years younger. Neither control group made many grammatical errors. The SLI group made substantially more grammatical errors in their written samples than in the spoken samples, with past tense omissions being the commonest form of error (affecting 26% of verbs in obligatory contexts).

### (b) Past tense inflections in SLI. Receptive language tasks

#### (i) Grammaticality judgement

A grammaticality judgement task—determining whether a sentence like ‘Yesterday John go to school’ is grammatical—can be used to test whether the child has syntactic knowledge of tense marking.

van der Lely & Ullman [29] compared 12 children with SLI aged 9–12 years with typically developing control groups of different ages, including one group who were 5 years younger and who performed at a comparable level on a test of sentence comprehension. The children with SLI were substantially more likely than controls to accept an ungrammatical finite sentence containing a verb stem. This contrasted with their responses to made-up irregular forms, for example ‘leck’ for ‘looked’, which they overwhelmingly rejected. In a similar vein, Rice *et al.* [30] presented children with a range of grammatical and ungrammatical sentences, including sentences with bare stems, for example ‘Maybe he need a Band-Aid’. Some sentences included additional information to indicate a third person singular context, e.g. ‘He look happy now’, and others involved omission of inflected copula or auxiliary (‘He brown’, ‘He running away’). These items were treated together as instances where the error involved failure to apply finite verb marking. Acceptance of grammatically erroneous forms was high relative to controls on these items, but not for other errors, such as failure of agreement (e.g. ‘I likes toast’) or dropping of -ing (‘He is run’). This pattern of results was replicated by Redmond & Rice [31], who studied processing of irregular verbs. They found 8-year olds with SLI were more likely than control 6-year olds to accept uninflected verbs in finite verb positions. Subsequently, Miller *et al.* [32] showed that 16-year olds with SLI were less sensitive than age-matched controls to grammaticality violation, including omission of tense markers. Overall, these results provide evidence that omission of inflectional endings by children with SLI cannot be attributed simply to problems at the stage of phonological output. Nor are difficulties confined to regular inflections.

#### (ii) Word monitoring

Montgomery & Leonard [33] used a reaction time task where children were required to respond as fast as possible to a target word in a heard sentence. The target word was preceded by a verb that was either inflected appropriately or was an (ungrammatical) bare stem. Control 6-year olds and 8-year olds responded more slowly when an obligatory inflection was omitted in the preceding verb. By contrast, 8-year olds with SLI were insensitive to this factor, despite showing good ability to detect ungrammaticality of similar items in an offline grammaticality judgement task (in contrast to other studies). Similar findings on a word-monitoring task were reported for 16-year olds with SLI: insensitivity to ungrammaticality was evident only when this involved omission of verb tense inflection [34].

### (c) Correlational evidence

SLI is seldom as specific as the label suggests and it is not uncommon to find co-occurring problems in non-linguistic domains, as well as a range of non-grammatical language deficits. It has been argued, however, that neither auditory impairments [35] nor non-verbal ability or receptive vocabulary [36] predict which children have problems with grammatical morphology. Phonological short-term memory (as assessed by nonword repetition) is a strong correlate of SLI, but there is only a weak relationship with omission of verb inflections [37].

## 5. Integrating the evidence

We shall now consider the evidence in relation to the model shown in figure 2, to see how far the errors made by children can be attributed to a deficit at a specific stage of processing.

### (a) Phonological formulation (step 4)

There is ample evidence that phonological complexity does impact on children's ability to produce past tense verbs [24,38], but phonological formulation problems cannot account for the range of deficits seen in SLI on tasks such as grammaticality judgement, word monitoring and written language. Nor can this account explain why production of the same phonological form is more accurate when it corresponds to a passive participle rather than a past tense verb [18].

### (b) Application of morpho-phonological rule (step 3)

There are several lines of evidence against a morpho-phonological explanation for tense errors in SLI. First, there is clear evidence for problems with irregular as well as regular past tense in elicitation tasks. Furthermore, children with SLI show some ability to apply a morphological rule, such as add -ed to a verb, as evidenced by their ability to inflect nonsense words and to overregularize irregular verbs. Finally, problems are not confined to tasks involving word production, but also occur on grammaticality judgement and word-monitoring tasks.

Could we nevertheless argue that complexity at the morpho-phonological level affects children's ability to process tense inflections? Sentence comprehension requires the child to parse incoming input in real time. If the child's language processor was unable to keep up with an incoming stream of words, then online syntactic interpretation might be disrupted, with inflections being particularly vulnerable to omission from a parsed representation [39]. This account, however, would predict insensitivity to inflections in general, and this is not seen [40]. Furthermore, adolescents with SLI showed normal reaction times in a word-monitoring task [34], contradicting the notion that they suffer from generally slowed receptive language processing.

### (c) Lexical entries for irregular forms (step 2)

This stage of processing is not generally regarded as a plausible explanation for tense errors in SLI, because knowledge of irregular verb inflections is not a specific source of difficulty, but is in line with general language ability [14].

### (d) Failure to assign grammatical feature of tense (step 1)

The pattern of errors observed in grammaticality judgement and word-monitoring tasks, plus the frequency of bare stem errors on irregular as well as regular verbs suggest that the problem for children with SLI is in knowing *when* to apply tense marking, rather than with *how* to do so. In other words, the problem is with syntax rather than morpho-phonological rules.

So, we are left with the question of why this aspect of grammar is disproportionately hard for children with SLI. Here, we find very different answers depending on the theoretical background of those proposing an account.

### (e) Domain-general learning accounts: perception, memory and statistical learning

As illustrated in the early stages of figure 1, to acquire language, the child must be able to distinguish salient features in the input and remember sequences of words. This raises the possibility that language acquisition could be disrupted by difficulty in perceiving, segmenting or remembering heard speech. Could such disruption be the root cause of problems with past tense? One piece of evidence that goes against such an explanation is the finding that children with mild-to-moderate hearing loss do not typically have the kinds of tense-marking problems that are seen in SLI, even though they do poorly on tests of speech perception and phonological short-term memory [20]. The weak relationship between tense-marking errors and poor phonological short-term memory (nonword repetition) in SLI is further evidence against this account [37].

### (f) A competence account: the extended optional infinitive

The difficulty of learning tense marking via local dependencies between words is one reason why generative linguists have argued for principles of Universal Grammar; normal learning mechanisms, it is argued, could not accomplish this feat, and so there must be innate knowledge of a grammatical feature such as tense.

According to a popular generative account, the child comes to the language-learning task armed with knowledge about the grammatical options available in different languages. Mastery of tense is achieved when exposure to language input triggers one setting or another of a parameter that specifies how tense is represented in that language. Triggering, however, implies a fairly abrupt transition from inaccurate to accurate tense usage, which is not what is seen in typical development. For a language such as English, the problem is dealt with by proposing that the distinction the child learns is between optional versus obligatory marking of tense in finite contexts [41].

To explain SLI, the proposal is that an optional setting for tense persists well beyond the usual age [42], hence this is known as the Extended Optional Infinitive account of SLI. Specifically, it is proposed that the parameter setting for tense undergoes maturation, and that this maturational process is delayed in some children—those with SLI.

An optionality account regards syntactic competence in SLI as immature. This is consistent with observations that these children go through a stage where they produce a mixture of correctly and incorrectly tensed verbs, but they seldom apply tense marking in appropriate syntactic contexts [40]. However, one piece of evidence is hard to accommodate within such a theory: children and adolescents with SLI perform far worse than controls when asked to judge sentences such as

*He made the robot fell into the pool,

where they tend to accept sentences with finite verbs as grammatical [31,32]. The theory predicts that finite or non-finite verbs will be seen in finite contexts, but only non-finite verbs should be seen in non-finite contexts, and so it has difficulty explaining such a result. It should, however, be noted that children's apparent insensitivity to this kind of grammatical error is unexpected, as they do not make similar errors in their own speech [31]. Furthermore, adolescents with SLI showed sensitivity to this kind of grammatical error on a word-monitoring task [30].

### (g) An explanation in terms of computational complexity

van der Lely [35] introduced the notion that at least some subtypes of SLI are due to deficits affecting syntactic, morphological or phonological computations. She argued that all these components of language are characterized by recursion and hierarchical non-local dependencies, and that problems with marking past tense could reflect difficulties at one or all of these levels. Although her account was derived from a generative perspective, the proposed problem is not with a specific parameter of the grammar, so much as in carrying out complex computations. Nevertheless, van der Lely argued that the underlying deficit was domain specific, insofar as it was not caused by a more general impairment in auditory or memory processing. A syntactic deficit in computational complexity would be expected to affect all aspects of grammar that required complex computations of nonlinear structure. As she noted, children with SLI do indeed have problems that extend beyond tense: they have difficulty understanding passives, producing questions and assigning pronominal reference as well.

### (h) An alternative domain-general performance account

According to van der Lely [35], computational operations, such as recursion or identification of hierarchical non-local dependencies, are quintessentially linguistic, and her postulated deficit in computational complexity is seen as domain specific. Such a view, however, has been challenged by Ullman & Pierpont [43], who have suggested that grammar is handled by a procedural learning system that is also implicated in sequential learning in other domains. According to this view, problems with tense marking could be due to a deficiency in a domain-general system that plays a key role in language learning and online language processing, but which is also involved in other tasks such as implicit learning of motor sequences. In recent years, several studies have found evidence compatible with this account, documenting deficiencies in non-verbal sequential motor learning in SLI [44–47]. Furthermore, although specific links with tense marking have not yet been demonstrated, some correlations have been documented between impaired motor sequence learning and grammatical abilities [44,45]. Such evidence suggests that there may be a domain-general sequence extraction mechanism that plays a role in online production and comprehension of complex language—similar to the computational complexity account of van der Lely [35], but not linguistically specific.

## 6. Implications for tense errors in acquired aphasia

One needs to be cautious of drawing parallels between acquired and developmental disorders [48]. Nevertheless, where similar phenomena are seen in children and adults, theories and methodologies can evolve quite separately, and it can be instructive to compare and contrast. For acquired disorders, the distinction between regular and irregular past tense verbs has been a major focus of attention and a great deal of debate has centred around the extent to which the Words and Rules account of language processing can account for the observed phenomena [10]. According to this account, there are two distinct systems used in past tense formation—one involves applying a rule to generate past tense from a base form and the other involves looking up an inflected form in the mental lexicon. Data from people with acquired language disorders have been used as evidence for this duality, on the basis that one can find instances of double dissociation: one group of people has problems predominantly with irregular inflection, whereas another group shows greater impairment with regulars. According to Pinker & Ullman [10], these correspond to deficits in the lexicon for the first group and the rule-based grammatical system for the second group. This interpretation has, however, been challenged by those who question the neat divide between two processing routes and who argue that phonological problems in production or perception could be responsible for the disproportionate problems with regular past tense seen in some people with aphasia [49].

The data from the cases reported by Ullman *et al.* [2] suggest, however, that those with anterior lesions, like children with SLI, may have problems in knowing *when* to mark tense. Data were reported for one case with a circumscribed frontal lesion, and five others with larger lesions. The prediction was that these individuals should perform worse at inflecting regular than irregular forms, should not overregularize and should be unable to apply past tense -ed to novel verbs. While it was true that the percentage of correctly inflected verbs was numerically greater for irregular than regular verbs, performance on irregular verbs was not impressive (55% correct for irregular and 20% for regular). For irregular as well as regular verbs, there were instances of unmarked forms (15% for irregular verbs versus 33% for regular verbs) or inappropriate application of the -ing inflection (6% of irregular verbs and 11% of regular verbs). This kind of evidence suggests that at least part of the failure to use correct inflections involves problems in understanding the syntactic context that obligates tense marking, rather than just knowing how to mark tense.

Similar points were made by Druks [50], in a case study of a man with Broca's aphasia and phonological dyslexia. Although on some tasks he made more errors on regular than irregular past tense verbs, his performance with irregular verbs was far from perfect and much worse than his performance with simple content words. Furthermore, performance with verb past tense inflections was worse than with noun inflections, derivational morphemes or present progressive -ing. Finally, the impairments with verb tense extended to comprehension and recognition tasks. The results of this case suggest that, as with SLI, an explanation in terms of syntactic impairment does a better job of explaining the pattern of errors than one that focuses only on the morpho-phonological or phonological levels.

The careful and painstaking research that has been conducted on SLI and acquired aphasia over the past two decades confirms that tense marking is often an area of particular difficulty. I have attempted to pull together the evidence from SLI and concluded that there is an underlying problem with syntax, rather than only with morphology or phonology. This is not to exclude a role of morphological or phonological deficits—it is quite likely that more than one linguistic system is affected and this may vary from child to child [35]. Insofar as tense is implicated, we need to recognize that an explanation in terms of syntax need not commit us to a generative view that attributes development to setting of a parameter of Universal Grammar. An explanation couched in terms of problems in extraction of hierarchical structure from sequential input may have a better chance of explaining the full pattern of linguistic strengths and weaknesses.

## References

[1] Rice ML 2000 Grammatical symptoms of specific language impairment. In Speech and language impairments in children: causes, characteristics, intervention and outcome (eds Bishop DVM Leonard LB), pp. 17–34. Hove, UK: Psychology Press.

[2] Ullman M-T Corkin S Coppola M Hickok G Growdon JH Koroshetz WJ Pinker S 1997 A neural dissociation within language: evidence that the mental dictionary is part of declarative memory, and that grammatical rules are processed by the procedural system. J. Cogn. Neurosci. 9, 266–276. (10.1162/jocn.1997.9.2.266) 23962016

[3] Edelman S Waterfall H 2007 Behavioral and computational aspects of language and its acquisition. Phys. Life Rev. 4, 253–277. (10.1016/j.plrev.2007.10.001)

[4] Tomasello M 2003 Constructing a language: a usage-based theory of language acquisition. Cambridge, MA: Harvard University Press.

[5] Rispoli M Hadley P 2010 Toward a theory of gradual morphosyntactic learning. In How children make linguistic generalizations: experience and variation in learning a first language (eds Arnon I Clark E). Amsterdam, The Netherlands: Benjamins.

[6] Leonard L 1989 Language learnability and specific language impairment in children. Appl. Psycholinguist. 10, 179–202. (10.1017/S0142716400008511)

[7] Joanisse MF Seidenberg MS 1998 Specific language impairment: a deficit in grammar or processing? Trends Cogn. Sci. 2, 240–247. (10.1016/S1364-6613(98)01186-3) 21244922

[8] Gopnik M Crago M 1991 Familial aggregation of a developmental language disorder. Cognition 39, 1–50. (10.1016/0010-0277(91)90058-C) 1934976

[9] Hsu HJ Bishop DVM 2010 Grammatical difficulties in children with specific language impairment (SLI): is learning deficient? Hum. Dev. 53, 264–277. (10.1159/000321289) PMC3191529 22003258

[10] Pinker S Ullman MT 2002 The past and future of the past tense. Trends Cogn. Sci. 6, 456–463. (10.1016/S1364-6613(02)01990-3) 12457895

[11] Seidenberg M Plaut DC In press Quasiregularity and its discontents: the legacy of the past tense debate. Cogn. Sci. 10.1111/cogs.12147 25104139

[12] Leonard LB 2007 Processing limitations and the grammatical profile of children with specific language impairment. In Advances in child development and behavior (ed. Kail RV), vol. 35, pp. 139–171. San Diego, CA: Elsevier Academic Press Inc. 10.1016/b978-0-12-009735-7.50009-8 17682325

[13] Rice ML Wexler K 2001 Rice/Wexler test of early grammatical impairment. London, UK: Psychological Corporation.

[14] Rice ML Wexler K Marquis J Hershberger S 2000 Acquisition of irregular past tense by children with specific language impairment. J. Speech Lang. Hear. Res. 43, 1126–1145. 11063235 10.1044/jslhr.4305.1126

[15] Marshall C van der Lely HKJ 2012 Irregular past tense forms in English: how data from children with specific language impairment contribute to models of morphology. Morphology 22, 121–141. (10.1007/s11525-011-9195-4)

[16] Leonard LB Deevy P Kurtz R Krantz Chorev L Owen A Polite E Elam D Finneran D 2007 Lexical aspect and the use of verb morphology by children with specific language impairment. J. Speech Lang. Hear. Res. 50, 759–777. (10.1044/1092-4388(2007/053)) 17538114

[17] Marshall CR van der Lely HKJ 2006 A challenge to current models of past tense inflection: the impact of phonotactics. Cognition 100, 302–320. (10.1016/j.cognition.2005.06.001) 16055110

[18] Leonard LB Deevy P Miller CA Rauf L Charest M Kurtz R 2003 Surface forms and grammatical functions: past tense and passive participle use by children with specific language impairment. J. Speech Lang. Hear. Res. 46, 43–55. (10.1044/1092-4388(2003/004)) 12647887

[19] van der Lely HKJ Ullman MT 2001 Past tense morphology in specifically language impaired and normally developing children. Lang. Cogn. Process. 16, 177–217. (10.1080/01690960042000076)

[20] Norbury CF Bishop DVM Briscoe J 2001 Production of English finite verb morphology: a comparison of SLI and mild-moderate hearing impairment. J. Speech Lang. Hear. Res. 44, 165–178. (10.1044/1092-4388(2001/015)) 11218100

[21] Clark HH 1973 The language-as-fixed-effect fallacy: a critique of language statistics in psychological research. J. Verbal Learn. Verbal Behav. 12, 335–359. (10.1016/S0022-5371(73)80014-3)

[22] Marchman VA Wulfeck B Ellis Weismer S 1999 Morphological productivity in children with normal language and SLI: a study of the English past tense. J. Speech Lang. Hear. Res. 42, 206–219. 10025555 10.1044/jslhr.4201.206

[23] Oetting JB Horohov JE 1997 Past-tense marking by children with and without specific language impairment. J. Speech Lang. Hear. Res. 40, 62–74. 9113859 10.1044/jslhr.4001.62

[24] Leonard LB Davis J Deevy P 2007 Phonotactic probability and past tense use by children with specific language impairment and their typically developing peers. Clin. Linguist. Phon. 21, 747–758. (10.1080/02699200701495473) 17882693 PMC4435720

[25] Leonard LB Eyer JA Bedore LM Grela BG 1997 Three accounts of the grammatical morpheme difficulties of English-speaking children with specific language impairment. J. Speech Lang. Hear. Res. 40, 741–753. 9263940 10.1044/jslhr.4004.741

[26] Slobin D Welsh CA 1973 Elicited imitation as a research tool in developmental psycholinguistics. In Studies of child language development (eds Ferguson CA Slobin D), pp. 485–497. New York, NY: Holt, Rinehart & Winston.

[27] Dalal RH Loeb DF 2005 Imitative production of regular past tense -ed by English-speaking children with specific language impairment. Int. J. Lang. Commun. Disord. 40, 67–82. (10.1080/13682820410001734163) 15832526

[28] Windsor J Scott CM Street CK 2000 Verb and noun morphology in the spoken and written language of children with language learning disabilities. J. Speech Lang. Hear. Res. 43, 1322–1336. 11193955 10.1044/jslhr.4306.1322

[29] van der Lely HKJ Ullman M 1996 The computation and representation of past-tense morphology in normally developing and specifically language impaired children. In 20th annual Boston University conference on language development (eds Stringfellow A Cahana-Amitay D Hughes E Zukowski A), pp. 792–803. Somerville, MA: Boston University, Cascadilla Press.

[30] Rice ML Wexler K Redmond SM 1999 Grammaticality judgments of an extended optional infinitive grammar: evidence from English-speaking children with specific language impairment. J. Speech Lang. Hear. Res. 42, 943–961. 10450913 10.1044/jslhr.4204.943

[31] Redmond SM Rice ML 2001 Detection of irregular verb violations by children with and without SLI. J. Speech Lang. Hear. Res. 44, 655–669. (10.1044/1092-4388(2001/053) 11407569

[32] Miller CA Leonard LB Finneran D 2008 Grammaticality judgements in adolescents with and without language impairment. Int. J. Lang. Commun. Disord. 43, 346–360. (10.1080/13682820701546813) 18446576 PMC2440708

[33] Montgomery JW Leonard LB 1998 Real-time inflectional processing by children with specific language impairment: effects of phonetic substance. J. Speech Lang. Hear. Res. 41, 1432–1443. 9859896 10.1044/jslhr.4106.1432

[34] Leonard LB Miller CA Finneran DA 2009 Grammatical morpheme effects on sentence processing by school-aged adolescents with specific language impairment. Lang. Cogn. Process. 24, 450–478. (10.1080/01690960802229649) PMC2727723 19690626

[35] van der Lely HKJ 2005 Domain-specific cognitive systems: insight from grammatical-SLI. Trends Cogn. Sci. 9, 53–59. (10.1016/j.tics.2004.12.002) 15668097

[36] Rice ML Tomblin JB Hoffman L Richman WA Marquis J 2004 Grammatical tense deficits in children with SLI and nonspecific language impairment: relationships with nonverbal IQ over time. J. Speech Lang. Hear. Res. 47, 816–833. (10.1044/1092-4388(2004/061)) 15324288

[37] Bishop DVM Adams CV Norbury CF 2006 Distinct genetic influences on grammar and phonological short-term memory deficits: evidence from 6-year-old twins. Genes Brain Behav. 5, 158–169. (10.1111/j.1601-183X.2005.00148.x) 16507007

[38] Marshall CM van der Lely H 2007 The impact of phonological complexity on past tense inflection in children with grammatical-SLI. Adv. Speech Lang. Pathol. 9, 191–203. (10.1080/14417040701261509)

[39] Bishop DVM 1997 Uncommon understanding: development and disorders of language comprehension in children. Hove, UK: Psychology Press.

[40] Bishop DVM 1994 Grammatical errors in specific language impairment: competence or performance limitation? Appl. Psycholinguist. 15, 507–549. (10.1017/S0142716400006895)

[41] Wexler K 1994 Optional Infinitives, head movement and the economy of derivations. In Verb movement (eds Lightfoot D Hornstein N), pp. 305–382. New York, NY: Cambridge University Press.

[42] Rice ML Wexler K Cleave PL 1995 Specific language impairment as a period of extended optional infinitive. J. Speech Hear. Res. 38, 850–863. 7474978 10.1044/jshr.3804.850

[43] Ullman MT Pierpont EI 2005 Specific language impairment is not specific to language: the procedural deficit hypothesis. Cortex 41, 399–433. (10.1016/S0010-9452(08)70276-4) 15871604

[44] Hedenius M 2011 Grammar predicts procedural learning and consolidation deficits in children with specific language impairment. Res. Dev. Disabil. 32, 2362–2375. (10.1016/j.ridd.2011.07.026) 21840165 PMC3191257

[45] Tomblin JB Mainela-Arnold E Zhang X 2007 Procedural learning in adolescents with and without specific language impairment. Lang. Learn. Dev. 3, 269–293. (10.1080/15475440701377477)

[46] Gabriel A Maillart C Stefaniak N Lejeune C Desmottes L Meulemans T 2013 Procedural learning in specific language impairment: effects of sequence complexity. J. Int. Neuropsychol. Soc. 19, 264–271. (10.1017/S1355617712001270) 23298411

[47] Hsu HJ Bishop DVM In press Sequence-specific procedural learning deficits in children with specific language impairment. Dev. Sci. 10.1111/desc.12125 PMC4031743 24410990

[48] Bishop DVM 1997 Cognitive neuropsychology and developmental disorders: uncomfortable bedfellows. Q. J. Exp. Psychol. 50A, 899–923. (10.1080/027249897391946) 9450382

[49] Bird H Lambon Ralph MA Seidenberg MS McClelland JL Patterson K 2003 Deficits in phonology and past-tense morphology: what's the connection? J. Mem. Lang. 48, 502–526. (10.1016/S0749-596X(02)00538-7)

[50] Druks J 2006 Morpho-syntactic and morpho-phonological deficits in the production of regularly and irregularly inflected verbs. Aphasiology 20, 993–1017. (10.1080/02687030600739422)

